# In the Vomiting of Pregnancy

**Published:** 1904-10

**Authors:** 


### IN THE VOMITING OF PREGNANCY.

Crowley, in the *Journal of the American Medical Association*, recommends this prescription for the vomiting of pregnancy:

| | | | |
|---|---|---|---|
| ℞ | Salicylate of bismuth | 4.00 | (ʒi.) |
| | Oxalate of cerium | 4.00 | (ʒi.) |
| | Menthol | .66 | (gr. x.) |
| | Muriate of cocaine | .19 | (gr. iii.) |
| | Bread | 4.00 | (ʒi.) |
| | Elixir of orange, to make | 180.00 | (℥vi.) |

Mix. Dose—Teaspoonful every three or four hours.

In many cases, however, it will be found unnecessary to use so much of a mixture as the above. Very often two or three drops of tincture of nux vomica in a few drops of water will control vomiting when everything else fails. This may be repeated every half hour. If it fails reduce the dose to one drop every ten minutes.

Another treatment which is deserving of trial is Francesco's. He uses valerian and reports two cases in which he was able to control the uterine reflexes which give so much trouble. The valerian was successful when other remedies, including morphine, both by mouth and rectum, had failed. The infusion is used as an enema.

